# A breeding strategy for high-oleic acid content and high-yield peanut using Tag SNPs and genomic selection

**DOI:** 10.1186/s12870-026-08473-2

**Published:** 2026-04-11

**Authors:** Minjie Guo, Jianli Miao, Yang Li, Junhua Yin, Peiyun Wang, Feng Luo, Shaowei Li, Junping Hu, Wenhao Liu, Taorui Zhang, Li Ren, Li Deng

**Affiliations:** 1 Peanut Institute, Kaifeng Academy of Agricultural and Forestry Sciences, Kaifeng, 475004 China; 2 Henan Seed Industry Development Center, Department of Agriculture and Rural Affairs of Henan Province, Zhengzhou, 450002 China

**Keywords:** Peanut, High-oleic, High yield, Genome-wide association study (GWAS), Tag SNPs, Genomic selection, Breeding

## Abstract

**Background:**

Developing peanut varieties with high oleic acid content (OAC) and superior yield is critical for meeting global nutritional and economic demands. To address this, our study integrated marker-assisted breeding with genomic selection (GS), creating an efficient breeding framework. Using a diverse natural population of 169 accessions, we conducted genome-wide association studies (GWAS) and GS analyses to identify Tag single nucleotide polymorphisms (SNPs) associated with OAC and develop a robust yield prediction model.

**Results:**

Phenotypic analysis indicated continuous variation in both OAC and productivity, with broad-sense heritability estimates of 0.9634 and 0.4535, respectively. Only a weak correlation was observed between these two traits. Whole-genome resequencing at approximately 10 × coverage identified 608,809 SNPs. GWAS revealed 32 significant loci associated with OAC, predominantly located on chromosomes 9 and 19, explaining 17.65–26.23% of the phenotypic variation. These loci were grouped into three distinct haplotype blocks, from which three core Tag SNPs (Arahy.9_113845844, Arahy.9_114322963, Arahy.19_154509990) were validated by regression and boxplot analyses. The GS model, developed using a genomic relationship matrix, yielded an additive genetic variance of 0.8626, a residual variance of 1.6915, a heritability estimate of 0.3377 for yield, with a prediction accuracy of 0.58. Validation in the candidate population showed optimal breeding efficiency at a 30% selection intensity using genomic estimated breeding values.

**Conclusions:**

The identified Tag SNPs provides a framework for efficient early-generation selection for OAC, while GS predictions facilitate advanced-generation yield optimization. Our results suggest that this integrated strategy has the potential to improve both quality and yield traits, offering a framework for more efficient breeding of peanut varieties with enhanced OAC and productivity.

**Supplementary Information:**

The online version contains supplementary material available at 10.1186/s12870-026-08473-2.

## Background

Peanuts are a globally significant oilseed crop, with kernels comprising approximately 50% oil by dry weight, of which oleic acid and linoleic acid constitute around 80% of total fatty acids [1, 37]. High-oleic peanut varieties (with oleic acid content, OAC ≥ 75%) exhibit superior oxidative stability, significantly enhancing the shelf life of peanut-derived products [46, 58]. Additionally, dietary intake of oleic acid has been well-documented for its beneficial effects on cardiometabolic health, thereby fueling increased commercial demand [16, 52]. Despite these benefits, conventional breeding methods often face challenges in simultaneously optimizing both OAC and yield, underscoring the urgency of developing peanut varieties that effectively integrate these two crucial traits.

The cultivated peanut (*Arachis hypogaea* L.), an allotetraploid (2n = 4x = 40, AABB), harbors OAC largely governed by two recessive, major-effect genes: *ahFAD2A* and *ahFAD2B* [12, 34]. In response, molecular markers such as Simple Sequence Repeat (SSR) and Kompetitive Allele-Specific PCR (KASP) targeting these loci have been developed and are widely implemented in high-oleic breeding programs [2, 68, 70]. However, accumulating evidence indicates that OAC behaves as a quantitatively inherited trait, displaying continuous variation and implicating the contribution of additional minor-effect loci [39, 51, 66]. For a more comprehensive and durable improvement of OAC traits, it is therefore imperative to uncover and integrate these subtle genetic contributors alongside known major-effect genes.

Genomic selection (GS), which utilizes genome-wide high-density single nucleotide polymorphism (SNP) markers to estimate breeding values by modeling the relationship between genomic and phenotypic data within a reference population [24, 44], has emerged as a transformative tool in modern animal and plant breeding [5, 23, 26, 49]. However, its application in allotetraploid peanuts remains unexplored, primarily due to the genomic complexity and historical lack of sense marker resources. Recent breakthroughs in sequencing the genomes of wild and cultivated peanut species have provided a solid foundation for developing high-resolution SNP datasets [7, 8, 11, 48, 67, 73]. By leveraging these genome-wide marker effects, thereby capturing the cumulative contributions of the entire genome and offering a powerful approach for accelerating genetic gain.

This study pioneers an integrated breeding system that combines marker-assisted selection (MAS) and GS for dual improvements in OAC and yield traits in peanut. We first performed genome-wide association analysis (GWAS) for OAC using a natural population comprising 169 core peanut accessions, identifying OAC-controlling loci and developing a set of Tag SNPs for precise early-generation detection of high-OAC genotypes during variety selection. Simultaneously, we established a GS prediction system for single-plant productivity (SPP) using the Genomic Best Linear Unbiased Prediction (GBLUP) model, enabling accurate evaluation of yield traits in advanced breeding generations.

The development of novel high-OAC and high-yield peanut varieties requires the simultaneous pyramiding of both OAC and yield traits, an outcome that remains difficult to achieve through conventional breeding alone. Advances in molecular biotechnology have made it possible to rapidly acquire genotype data across breeding materials. By utilizing Tag SNPs for early-generation selection of high-OAC genotypes in the F_2_ population and applying GS models to predict yield potential in advanced generations, we propose an efficient dual-trait pyramiding strategy. This approach is expected to significantly accelerate the breeding cycle and enhance the precision and success rate of developing superior peanut cultivars.

## Materials and methods

### Plant materials and field trial design

All 169 accessions used in this study were derived from “Kaixuan 016” (Ren et al., 2015) as a direct or indirect common core parent, and were developed through hybridization or backcrossing with diverse parental resources from different institutions, including local landraces, introduced lines, and mutant materials (Supplementary Dataset S1): 154 from the Kaifeng Academy of Agricultural and Forestry Sciences in Henan Province (China), 12 donated by the Institute of Cereal and Oil Crops, Hebei Academy of Agricultural and Forestry Sciences in Shijiazhuang (China), two donated by the Oil Crops Research Institute, Chinese Academy of Agricultural Sciences in Wuhan (China), and one donated by Agricultural Consulting, Inc. in Georgie, USA. From these, 20 accessions were randomly selected as a validation population for GWAS and GS analyses (Supplementary Dataset S2). Multi-environment trials were conducted at four locations in Henan Province across three years (E1: Kaifeng 2019; E2: Xinyang 2019; E3: Kaifeng 2020; E4: Kaifeng 2021) using a randomized complete block design with three replicates [20]. All trials followed standardized cultivation protocols: ridge planting with double rows (1.6 m × 8.4 m plot size, 13.44 m^2^ per accession), sown around May 10th with two seeds per hill at 20 cm spacing within rows and 40 cm between rows, and harvested around September 15th annually.

### Phenotypic evaluation

Single-plant productivity (SPP) was evaluated using a randomized complete block design across four distinct environments, with three replicates per location. After peanut plants reached full maturity, harvesting was conducted sequentially according to their developmental stage. Within each replicate plot, ten consecutive plants were selected from the central area of the plot that exhibited normal growth, with no missing plants and no visible disease or pest damage, and their pods were harvested. The harvested pods were air-dried under well-ventilated conditions until a constant weight was achieved (kernel moisture content < 10%). The total pod weight of the ten plants was then measured, and the average value was calculated to represent SPP for that replicate.

Oleic acid content (OAC) of peanut kernels was determined using a Perten DA7250 near-infrared (NIR) spectrometer (Perten Instruments, Sweden). The instrument was equipped with a dedicated fatty acid prediction model developed from a large peanut germplasm dataset precisely calibrated using gas chromatography. To ensure measurement accuracy for each batch, the model was calibrated prior to analysis using peanut standard samples with known chemical values representing high, medium, and low oleic acid contents. After calibration validation, intact, mold-free, and well-filled kernels dried from each plot were loaded into the specialized sample cup, leveled, and subjected to three independent consecutive scanning measurements.

### DNA extraction and sequencing

Approximately 200 mg of fresh leaf tissue was collected from each of the 169 peanut accessions at the seedling stage for genomic DNA extraction, following the cetyltrimethylammonium bromide (CTAB) protocol described by Doyle [18]. The integrity and purity of the extracted DNA were assessed via 1% agarose gel electrophoresis and quantified using a NanoDrop 2000 spectrophotometer (Thermo Fisher Scientific, Waltham, MA, USA). High-quality DNA samples were subsequently submitted to Novogene Bioinformatics Technology Co., Ltd. (Beijing, China) for whole-genome resequencing at 10 × coverage using the Illumina HiSeqTM 2000 platform (Illumina, San Diego, CA, USA). Raw image files were processed through base calling to generate FASTQ-format sequence data. Following rigorous quality control and adapter trimming, high-quality clean reads were retained for downstream genomic analyses.

### SNP alignment and calling

Prior to alignment, adapter sequences and low-quality paired-end reads were removed to ensure data integrity. The resulting high-quality reads were aligned to the *A. hypogaea* reference genome cultivar Kaixuan 016 [48] using the MEM algorithm implemented in BWA (Burrows-Wheeler Aligner) software [36], generating SAM-format alignment files. These were subsequently converted into sorted BAM files using SAMtools, and PCR duplicates were eliminated using the 'rmdup' command [38]. Variant calling for each accession was performed using the HaplotypeCaller module of the Genome Analysis Toolkit (GATK, [43]), generating individual gVCF files. Final SNP identification at the population level was demonstrated through joint genotyping of all gVCFs using the GATK GenotypeGVCFs function, enabling comprehensive detection of SNPs across the full panel of accessions.

### Data analysis

Phenotypic data were initially processed using Microsoft Excel 2010, with outliers identified and treated as missing values. Variance components for oleic acid content (OAC) and single-plant productivity (SPP) were estimated using a linear mixed model implemented in ASReml [22]. In the model, environments and replicates nested within environments were treated as fixed effects, whereas genotypes and genotype × environment (G × E) interactions were treated as random effects. Broad-sense heritability (h^2^) for OAC and SPP was calculated according to the following formula: h^2^ = σ^2^_g_/(σ^2^_g_ + σ^2^_ge_/n + σ^2^_ԑ_/nr), where σ^2^_g_ represents the genotypic variance, σ^2^_ge_ represents the variance due to genotype x environment interaction,σ^2^_ԑ_ denotes the residual variance component; n is the number of environment trials; and r is the number of replicates in each environment trial [29]. Best linear unbiased estimates (BLUEs) for OAC and SPP across four environments were obtained using a mixed linear model implemented in Genstat® version 23 [61].

Genotypic data from the candidate population were integrated with the reference dataset to construct a comprehensive genotypic matrix. Rigorous quality control measures were applied by using PLINK v1.9, including the exclusion of samples and loci exhibiting greater than 10% missing data and removal of loci with a minor allele frequency (MAF) below 1%, ensuring a high-confidence dataset for subsequent genetic analyses.

GWAS were performed by integrating phenotypic and genotypic datasets using a mixed linear model (MLM) framework [72] to account for population structure and kinship. For loci exhibiting significant associations, the proportion of phenotypic variance explained (PVE) was quantified, and linkage disequilibrium (LD) block analysis was subsequently performed using PLINK software [17, 65]. Statistical power for significant loci was evaluated using a non-central chi-squared approach as described by Visscher et al. [60]. The non-centrality parameter (NCP) was first calculated using the formula:

$$\mathrm{NCP}=\mathrm{n}\times \frac{{\mathrm{R}}^{2}}{(1-{\mathrm{R}}^{2})}$$

where n represents the sample size and R^2^ denotes the proportion of phenotypic variance explained (PVE) by the SNP. Power for each significant SNP was then calculated in R using the qchisq and pchisq functions, based on the GWAS significance threshold of 1e-7 and the corresponding NCP value. Tag SNPs were identified using PLINK v1.9 with parameters (–tag-r2 0.8, –tag-kb 250 kb), enabling the selection of representative SNPs with the highest pairwise LD (r^2^) within each defined genomic block [33, 55]. The predictive power of the selected Tag SNPs was evaluated via multiple linear regression using the “lm” function in R [27]. Trait-genotype associations were visualized through boxplots generated with the ggplot2 package [64], and statistical significance was assessed using Student’s *t*-test.

After quality control, GS workflow included the following steps: (1) construction of the genomic relationship matrix (G) using the filtered SNP dataset. The G matrix was calculated in the R environment using the ASRgenomics package [21]. G used in GS was constructed following the method of VanRaden [59], using the formula:

$$G=\frac{ZZ^\prime}{2\sum\rho_i\left(1-\rho_i\right)}$$

where *p*_*i*_ represents MAF at the *i*-th locus, Z is the centered SNP genotype matrix, and Z’ denotes its transpose. The formulation captures the realized genomic relationship among individuals based on marker data.

(2) The GBLUP model was fitted using the ASReml-R software to estimate genomic estimated breeding values (GEBVs) for individual genotypes. The model was specified as follows:

$$\left[\begin{array}{cccccccccccc} \widehat b\\ \widehat u\end{array}\right]=\left[\begin{array}{cccccccccccc} X^\prime X&X^ \prime Z\\ Z^\prime X&Z^\prime Z+\text G^{-1}\text k\end{array}\right]^{-1}\left[\begin{array}{cccccccccccc} X^\prime Y\\ Z^\prime Y\end{array}\right]$$

where X represents the design matrix for fixed effects, Z denotes the design matrix for random genetic effects, and Y is the matrix of observed phenotypic values. ${G}^{-1}$ constitutes the inverse of the genomic relationship matrix, $\widehat{b}$ signifies the fixed effect estimates (BLUE), $\widehat{u}$ indicates the random effect estimates (BLUP), and k represents the ratio of residual variance to additive genetic variance, ensuring partitioning of genetic and environmental effect in GEBV prediction.

(3) Model performance was evaluated using five-fold cross-validation implemented in R with the car package [19]. Model performance was assessed using two metrics: predictive ability and prediction accuracy [32]. Predictive ability was defined as the Pearson correlation coefficient between the predicted values and the adjusted phenotypic values, calculated as follows:

$$\text r=\mathrm{COR}\left(\mathrm{GEBV}, \mathrm{Yc}\right)$$

In the above equation, COR denotes the Pearson correlation coefficient; Yc represents the adjusted phenotypic value; and GEBV refers to the genomic estimated breeding value.

Prediction accuracy was obtained by standardizing predictive ability by the heritability of the trait and was calculated as follows:

$$\mathrm{Accuracy}=\frac{r_{\left(gy\right)}}{\sqrt{h^2}}$$

where r denotes predictive ability, g represents the GEBV, y denotes the phenotypic value, and h^2^ represents the heritability of the trait.

## Results

### Phenotypic and SNP variation analysis

**Fig. 1:**
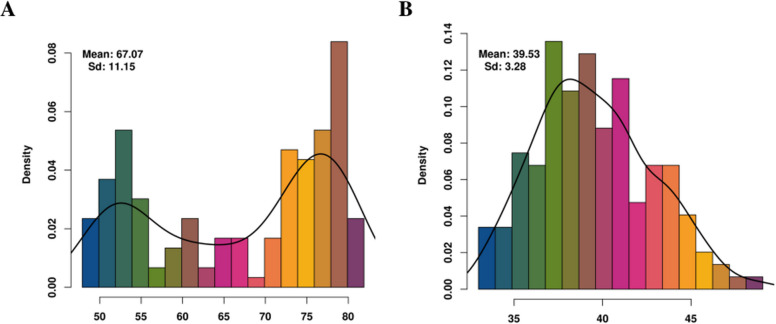
Phenotypic variation histogram of oleic acid content (OAC) and single-plant productivity (SPP). **A** Histogram depicting the distribution of best linear unbiased estimates (BLUEs) for OAC, displaying a bimodal pattern across accession. **B** Histogram of BLUE values for SPP, exhibiting a near-normal distribution characteristic of a quantitative trait

Analysis of BLUE for OAC and SPP across four environments revealed distinct phenotypic patterns. OAC exhibited a pronounced bimodal distribution (Fig. 1A), with the first peak corresponding to low-OAC accessions (OAC < 65%) and the second peak centered around high-OAC genotypes (OAC ≈ 75%). Notably, both subpopulations displayed continuous variation, indicating the presence of underlying polygenic regulation within each group. Across the four environments, mean OAC values ranged from 63.43% to 70.23%. The high broad-sense heritability (H^2^ = 0.9634; Table 1) underscores the trait’s strong genetic control and highlights its suitability for early-generation selection in breeding programs. In addition, a combined ANOVA was conducted for the multi-environment data (Supplementary Dataset S3). Although the genotype × environment (G × E) interaction was statistically significant, the mean square of the interaction effect accounted for only 0.81% of the genotypic main-effect mean square (12/1486), indicating that its overall impact was relatively limited. SPP exhibited a near-normal distribution (Fig. 1B), consistent with the characteristics of a quantitative trait controlled by numerous loci of minor effect. Mean SPP values, measured in three uncontaminated environments, ranged from 32.82% to 45.92%. The relatively low broad-sense heritability estimate (H^2^ = 0.4535) indicates a substantial influence of environmental factors. Correlation analyses of phenotypic data for OAC and SPP across three environments revealed only weak associations between the two traits, with correlation coefficients of 0.02, − 0.09, and − 0.11, none of which reached statistical significance (Supplementary Dataset S4).

**Table 1:** Analysis of variations in the oleic acid content (OAC) and single-plant productivity (SPP) of 169 peanut accessions grown under different environments

Trait	Environment	Max	Min	Mean	Variance	SD	CV (%)	H^2^
OAC	E1	84.78	39.96	66.35	173.47	13.17	19.85	0.9634
	E2	88.15	44.45	70.23	165.30	12.86	18.31	
	E3	79.56	42.86	63.43	117.64	10.85	17.10	
	E4	80.74	39.56	64.28	175.53	13.25	20.61	
SPP	E1	60.27	33.50	45.92	28.09	5.30	11.54	0.4535
	E2	50.10	23.76	32.82	15.51	3.94	12.00	
	E3	46.92	22.59	34.58	18.94	4.35	12.59	

*OAC* Oleic acid content, *SPP* Single-plant productivity, *SD* Standard deviation, *CV* Coefficients of variation, *H*^*2*^ Broad-sense heritability, *E* Environment

**Fig. 2:**
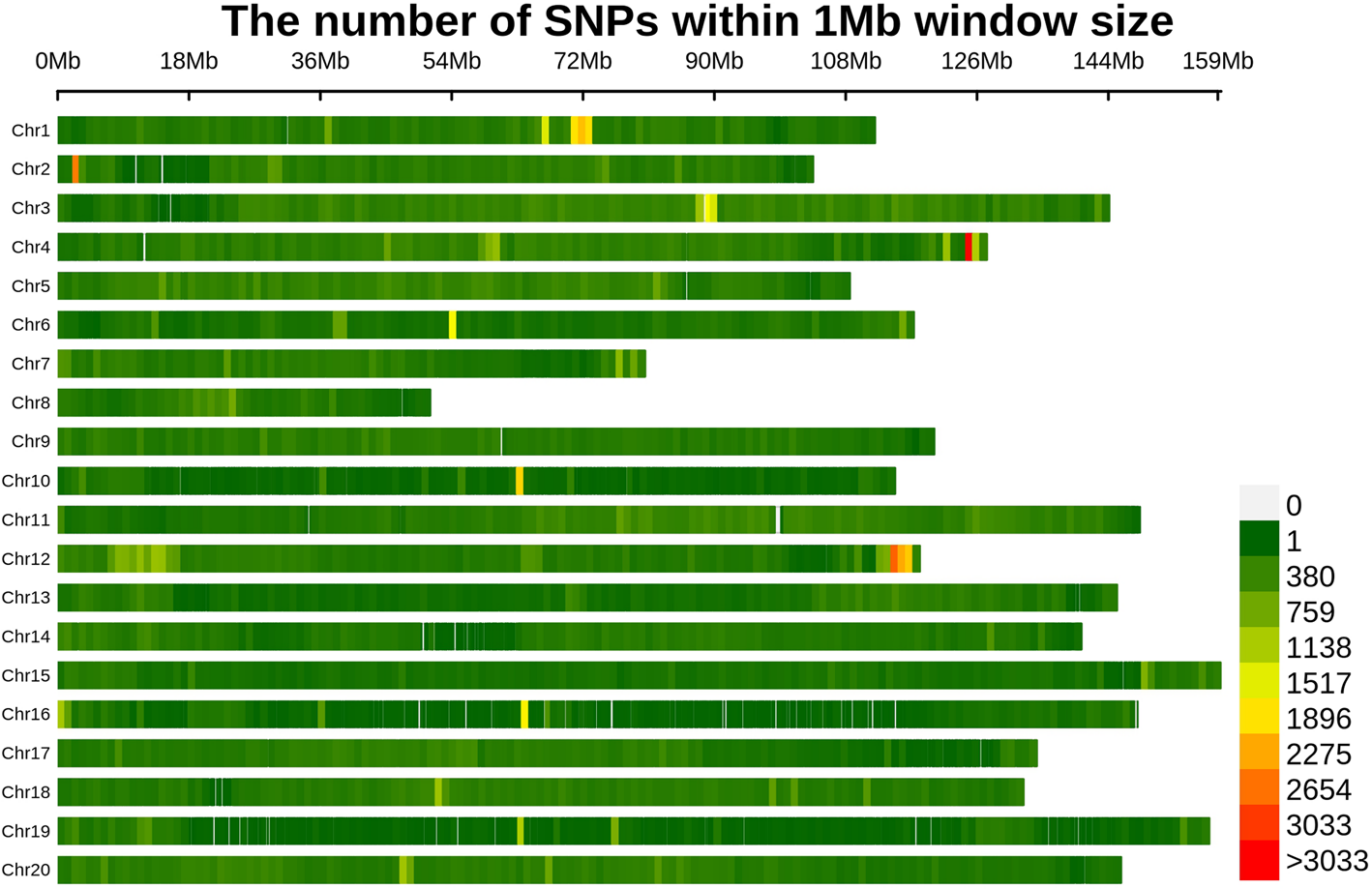
Genome-wide single-nucleotide polymorphism (SNP) distribution in peanut panel. Genome-wide SNP density heatmap showing the number of SNPs within 1 Mb sliding windows across the 20 peanut chromosomes. SNP density ranges from 0 to over 3,033 SNPs per window, with color intensity increasing from green to red to represent higher SNP density

Following stringent quality control filtering, a total of 608,809 high-confidence SNPs were retained across the genome. Chromosomal distribution analysis revealed substantial variation in SNP density, with Chromosome 03 (Chr03) exhibiting the highest SNP density (48,821 SNPs) and Chr08 the lowest (13,143 SNPs). The average genome-wide SNP density was calculated at 242.56 SNPs per megabase (Mb) (Fig. 2). The high sequencing depth and coverage across all accessions ensured the resulting genotypic dataset met rigorous accuracy standards, providing a robust foundation for downstream genomic analysis.

### Identification of significant SNPs for OAC

**Fig. 3:**
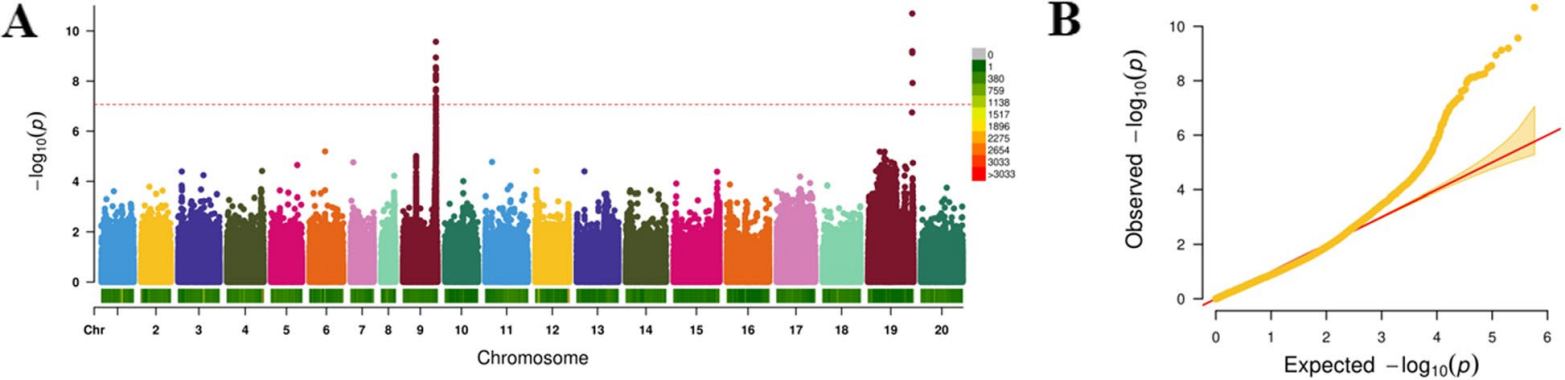
Genome-wide association analysis for oleic acid content (OAC) across 149 peanut accessions. **A** Manhattan plot displaying -log_10_(*P*) values for single-nucleotide polymorphism (SNP) associations with OAC across all 20 chromosomes. The horizontal dashed line indicates the Bonferroni-corrected significance threshold (-log_10_(*P*) = 7.0). Notable association peaks are observed on chromosomes 9 and 19. **B** Quantile–quantile (Q-Q) plot showing the distribution of observed versus expected -log_10_(*P*) values for OAC, indicating significant deviations from the null hypothesis and suggesting true genetic association

GWAS analysis was performed using combined genotype and phenotype data from 149 accessions (Fig. 3). A stringent significance threshold of -log_10_(*P*) ≥ 7.0 was applied based on Bonferroni correction (α = 0.05/N, where N represents the total number of SNPs) to identify SNPs significantly associated with OAC. The Manhattan plot (Fig. 3A) and quantile–quantile (QQ) plot (Fig. 3B) demonstrated a clear deviation from the expected distribution under the null hypothesis, indicating strong genetic signals. Notably, the majority of significant loci were clustered on Chr9 and Chr19 (Table 2), with a total of 32 SNPs surpassing the significance threshold, 28 located on Chr9 and 4 on Chr19, highlighting these regions as key genomic hotspots potentially governing OAC variation.

**Table 2:** Analysis of variations in the oleic acid content (OAC) and single-plant productivity (SPP) of 169 peanut accessions grown under different environments

Block	Chr	SNP sites								Tag SNPs
A	9	113,845,844	113,858,071	113,858,669	113,874,096	113,898,323	113,899,092	113,918,792	113,940,661	113,845,844
		113,951,897	113,962,231	113,963,333	113,985,040	114,001,925	114,002,898	114,035,070	114,046,758	
		114,091,718	114,100,766	114,167,704	114,183,334	/	/	/	/	
B	9	114,195,794	114,241,585	114,255,210	114,261,850	114,322,963	114,323,009	114,325,078	114,378,317	114,322,963
C	19	153,409,182	153,491,111	153,598,939	154,509,990	/	/	/	/	154,509,990

To evaluate the phenotypic effects of the identified allelic variants, the PVE was calculated for all 32 significant SNPs. These loci collectively accounted for 17.65–26.23% of the observed phenotypic variation in OAC (Supplementary Dataset S5). Power analysis revealed that the statistical power of these significant SNPs ranged from 0.63 to 0.97, with a mean value of 0.80, indicating that the detected associations and their corresponding PVE estimates are robust and reliable. These relatively high PVE values indicate that these genomic regions exert substantial influence on trait expression, likely encompassing key functional genes or regulatory elements involved in oleic acid biosynthesis and accumulation in peanut.

### Mining of Tag SNPs

**Fig. 4:**
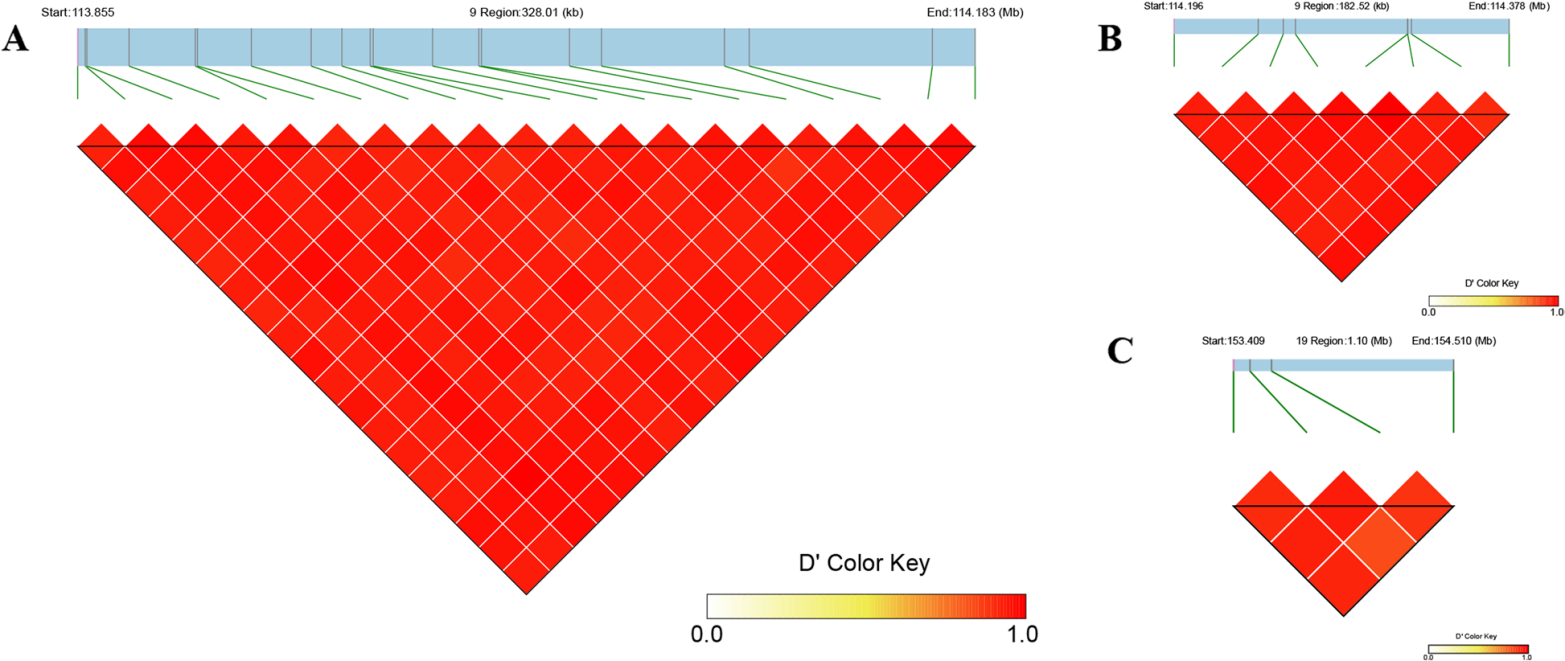
Linkage disequilibrium (LD) structure of genomic regions containing significant single nucleotide polymorphisms (SNPs) associated with oleic acid content (OAC). Blocks A, B, and C represent three distinct haplotype blocks identified on chromosomes 9 and 19. The heatmap illustrates pairwise LD between SNPs, with color intensity reflecting the magnitude of DL as measured by D'. Higher D' values indicate stronger correlation and tighter linkage between SNP pairs, suggesting regions of limited historical recombination and potential co-inheritance of alleles

In light of the clustering of significant loci on Chr9 and Chr19 and their observed LD, Tag SNPs were developed to enhance the efficiency of MAS for OAC improvement. Genotypic data from the 32 significant loci across 169 peanut accessions were subjected to LD block analysis, revealing the presence of three distinct haplotype blocks comprising 8, 20, and 4 SNPs, respectively (Table 2; Fig. 4). These Tag SNPs represent key markers with strong predictive power for OAC and hold considerable potential for integration into breeding pipelines targeting high-OAC peanut cultivars.

To optimize the SNP marker set for MAS, a single representative Tag SNP was selected from each haplotype block, thereby streamlining the marker panel without compromising predictive power. LD patterns were analyzed within 500 kb genomic windows, and the SNP with the highest mean *r*^*2*^ value in each block, representing the strongest co-segregation with neighboring SNPs, was chosen. As a result, three Tag SNPs were identified: Arahy.9_113845844 (Block 1), Arahy.9_114322963 (Block 2), and Arahy.19_154509990 (Block 3) (Table 2, Fig. 4). These markers constitute a refined panel for efficient selection of high-OAC genotypes in peanut breeding programs.

### Verification of Tag SNPs

**Fig. 5:**
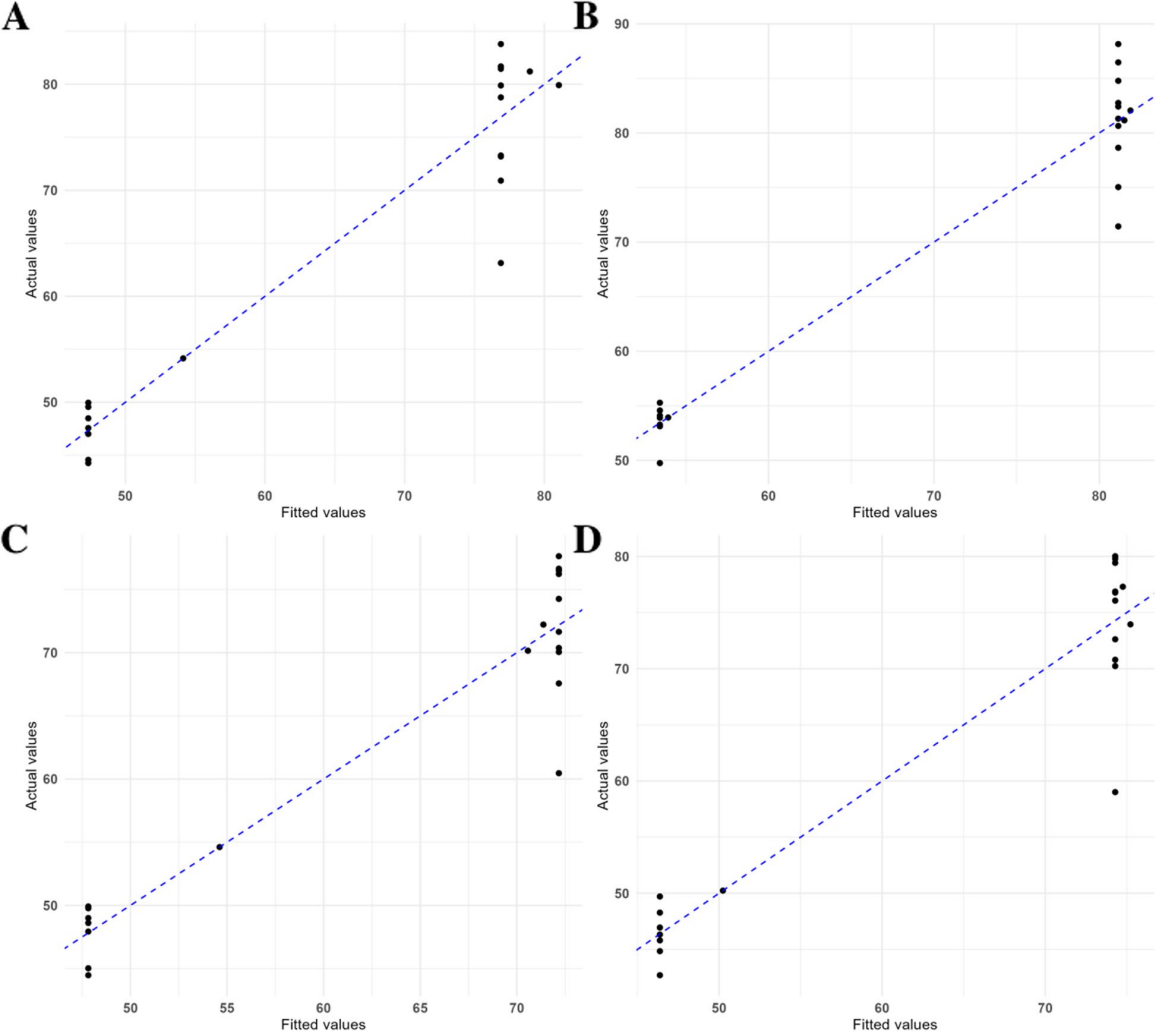
Validation of the regression model for selected Tag single-nucleotide polymorphisms (SNPs) in four environment. Scatterplot illustrates the relationship between predicted and observed oleic acid content (OAC) values in 20 peanut accessions based on a multivariance model using three Tag SNPs. The dashed blue line represents the regression fit, with an adjusted R^2^ of 0.8921, 0.9242, 0.8845, 0.8904, indicating high predictive accuracy and strong concordance between observed and model-estimated phenotypic values

To validate the predictive utility of the selected Tag SNPs, genotypic data from three representative loci were analyzed across 20 candidate accessions (Supplementary Dataset S2). Genotypes were numerically encoded as follows: 0 for major allele homozygotes, 1 for heterozygotes, and 2 for minor allele homozygotes, serving as explanatory variables (X). Field-derived, adjusted phenotypic values were used as the response variable (Y). The regression analysis yielded an adjusted R^2^ of 0.9074, Using multi-year, multi-environment phenotypic data, best linear unbiased estimates (BLUEs) were calculated and used to validate marker performance within each environment. As shown in Fig. 5, these markers consistently exhibited strong performance across four independent environments, with adjusted R^2^ values of 0.8921, 0.9242, 0.8845, and 0.8904, respectively. The close agreement between observed (y-axis) and predicted (x-axis) values indicates a high level of explanatory power, suggesting that these three loci represent stable candidate markers across multiple environments, though broader validation is still needed.

**Fig. 6:**
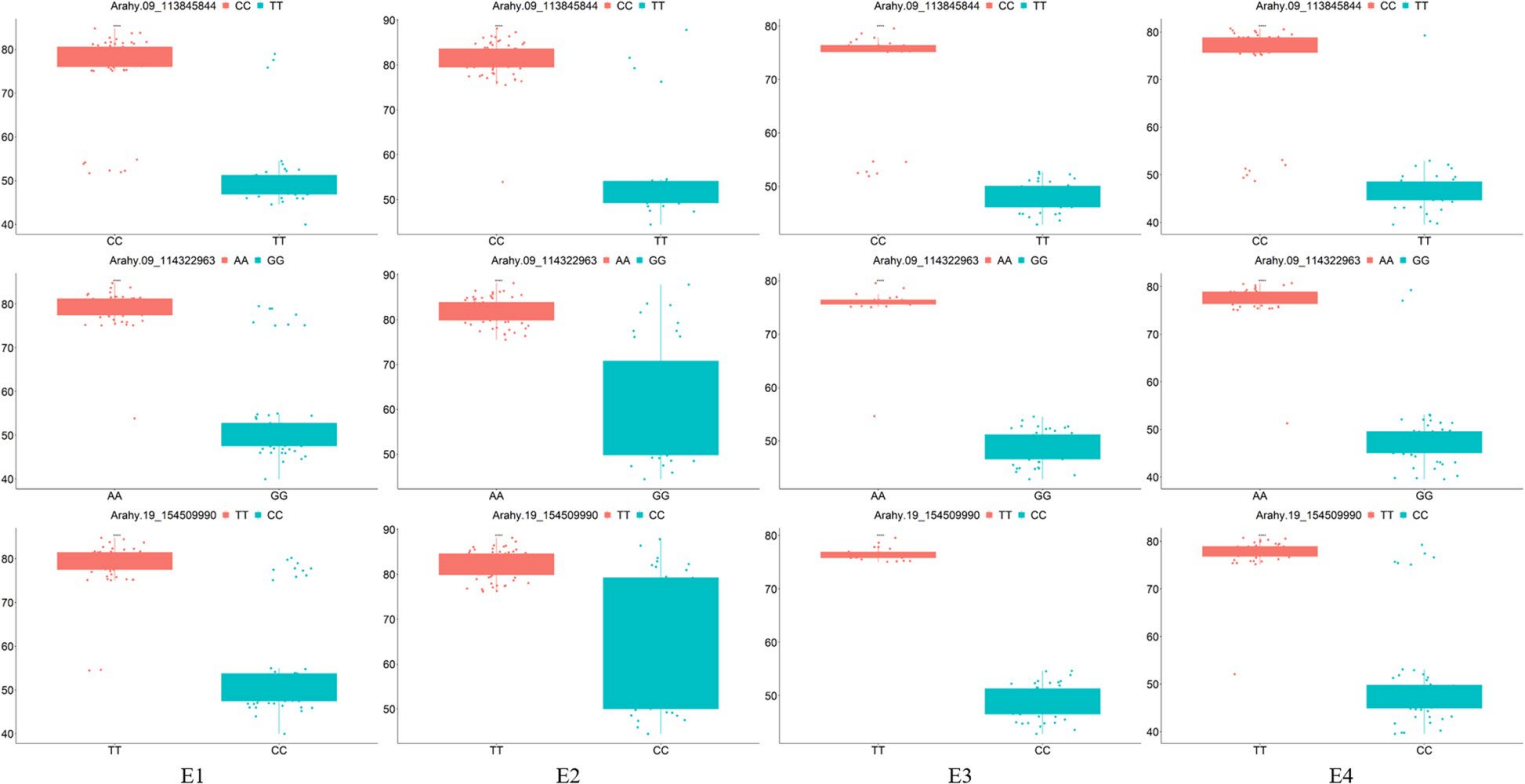
Phenotypic variation in oleic acid content (OAC) across genotypes at three Tag single-nucleotide polymorphism (SNP) loci. Boxplots display the distribution of OAC for different allelic groups at three validated Tag SNPs (Arahy.09_113495644, Arahy.09_114322963, and Arahy.19_154509990) across four environments: E1 (Kaifeng in 2019), E2 (Xinyang in 2019), E3 (Kaifeng in 2020), E4 (Kaifeng in 2021). Each panel compares the phenotypic values between two genotypic classes, highlighting consistent and significant differences across environments

To further validate the discriminatory power of the selected Tag SNPs, genotype data for the three loci, Arahy.9_113845844, Arahy.9_114322963, and Arahy.19_154509990, were extracted from all 169 accessions (Supplementary Dataset S6). A subset of 50 high-OAC accessions (OAC ≥ 76.79%) and 50 low-OAC accessions (OAC ≤ 54.85%) were selected for boxplot validation based on their phenotypic values (Fig. 6). The resulting boxplot revealed clear phenotypic stratification across genotypes at each SNP locus (Fig. 6). At Arahy.9_113845844, individuals with the CC genotype consistently exhibited high-OAC levels, while the TT genotype was predominantly associated with low-OAC. Similarly, at Arahy.9_114322963, the AA genotype corresponded to high-OAC phenotypes, whereas the GG genotype was linked to low-OAC. At Arahy.19_154509990, the TT genotype was indicative of high-OAC, while the CC genotype aligned with low-OAC values. These patterns underscore the effectiveness of the selected SNPs as diagnostic markers for oleic acid content and their potential utility in marker-assisted breeding programs.

### GS model construction

A standardized genomic relationship matrix (G-matrix) was constructed using quality-filtered genotypic data from all 169 accessions. The GBLUP model was trained in a reference population comprising 149 accessions with both genotype data and BLUE values for SPP. An independent prediction set of 20 accessions (Supplementary Dataset S2), possessing genotypic data only, was used to validate by comparing predicted GEBVs with observed phenotypic values. The GBLUP model was implemented using a restricted maximum likelihood (REML) approach, incorporating both the G-matrix and phenotypic data to estimate genetic parameters and GEBVs for SPP (Supplementary Dataset S7). Key model performance indicators included the estimated additive genetic variance (0.8626), residual variance (1.6915), and H^2^ (0.3377) (Supplementary Dataset S8). Genomic prediction performance was evaluated using five-fold cross-validation with 20 repetitions, resulting in 100 different training and test set combinations. This procedure yielded a predictive ability of 0.3320, corresponding to a standardized genomic prediction accuracy of 0.58. These values fall within expected theoretical ranges, indicating proper model convergence and supporting the reliability and robustness of the GS approach for predicting complex traits such as SPP.

### GS model validation

**Fig. 7:**
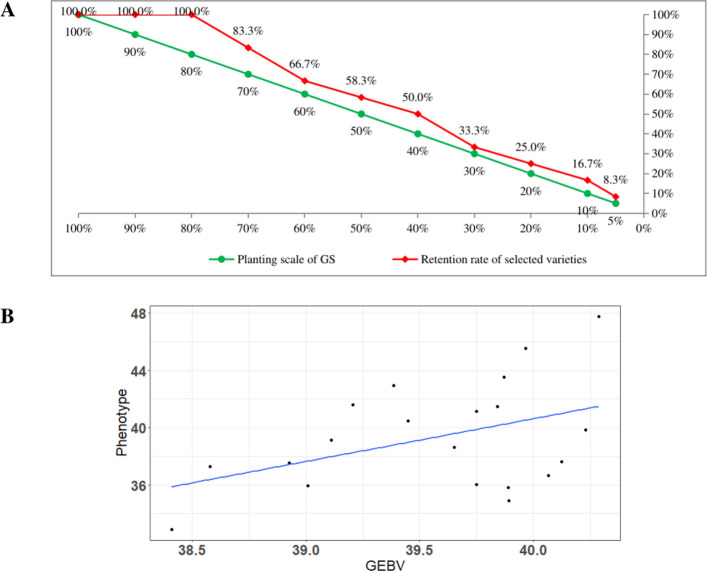
Accuracy assessment of genomic selection (GS) in peanut breeding. **A** Relationship between the planting scale of GS (green line) and elite variety advancement retention rate (red line) across varying selection intensities. As selection pressure increases (lower planting scale), the proportion of retained elite varieties declines, illustrating the trade-off between selection stringency and genetic grain. **B** Scatterplot illustrating the correlation between genomic estimated breeding values (GEBVs) and observed phenotypic values. The fitted regression line (blue) indicates a positive association, supporting the predictive accuracy of the GS model

To assess the predictive performance of the GS model, we conducted a validation using a candidate population of 20 peanut accessions. GEBVs were compared against observed phenotypic data for SPP. For practical breeding applications, the top 12 accessions, ranked according to phenotypic SPP and their breeding retention value, were designated as elite varieties. The model’s utility was further evaluated under scenarios simulating reductions in trial size (Fig. 7A, Supplementary Dataset S9). Remarkably, a 20% reduction retained all elite accessions (100%, 12/12), whereas reductions of 30%, 40%, and 50% resulted in retention rates of 83.3% (10/12), 66.7% (8/12), and 58.3% (7/12), respectively. These results demonstrate that reducing the trial size by up to 30% achieves an optimal balance, sustaining high selection efficiency (exceeding 80%) while significantly reducing field trial costs. The predictive fidelity of the GS model was quantitatively supported by a Pearson correlation coefficient of 0.4292 (P < 0.05) between GEBVs and phenotypic BLUEs [28]. The clustering of data points along the identity line (Fig. 7B, Supplementary Dataset S9) further attests to the model’s reliability in capturing phenotypic variation, thus meeting the expected standards for GS implementation in breeding programs.

### Application of Tag SNPs and GS in breeding programs

**Fig. 8:**
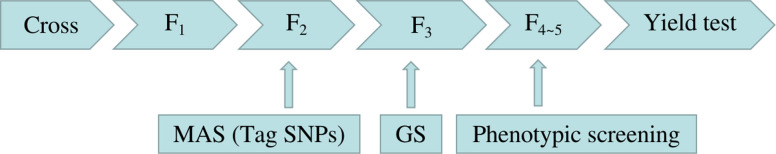
Schematic overview of the breeding pipeline for developing high-oleic acid content (OAC) and high-yield peanut varieties. The diagram illustrates the integrated workflow combining phenotypic evaluation, genome-wide association studies (GWAS), Tag single-nucleotide polymorphism (SNPs) selection, and genomic selection (GS). MAS, molecular marker-assisted selection

To develop peanut varieties with both high-OAC and high-yield through pedigree selection, we propose a streamlined breeding pipeline that synergistically integrates Tag SNP-based MAS with GS (Fig. 8). The proposed protocol comprises the following sequential steps. (1) Crosses are initiated between high-yield lines and high-OAC lines, and true F_1_ hybrids are confirmed through genetic authentication. (2) In the F_2_ generation, whole-plant DNA is collected for early screening. Plants with poor yield performance are first eliminated based on phenotypic evaluation, and the remaining individuals are genotyped using three validated Tag SNPs to identify plants that simultaneously meet the selection threshold for OAC and yield. (3) F₃ families derived from selected F_2_ individuals are subjected to GS. At the seedling stage, whole-plant DNA of the F_3_ plants is archived. Following phenotypic selection at harvest, elite individuals are re-sequenced and their GEBVs for SPP are predicted using the established reference GS model. (4) F_4~5_ families with superior GS-selected performance are further evaluated through field-based phenotypic screening, focusing on key agronomic traits such as plant architecture, stress resilience, and yield components. (5) After the F_5_ generation, advanced lines enter multi-environment yield trails, from which stable elite lines with superior generation performance are ultimately selected. This integrated breeding strategy, which combines MAS and GS within a single pipeline, is expected to substantially improve selection efficiency and accelerate the development of high-OAC, high-yield peanut cultivars.

## Discussion

The development of high-OAC and high-yield peanut varieties is important for meeting the specialized, value-added demands of the processing industry and for advancing sustainable, high-quality agricultural. In this study, we integrated GWAS and GS technologies to identify key genetic loci associated with OAC in peanut. Building on these findings, we proposed a genomic-informed breeding model aimed at supporting the simultaneous enhancement of yield and oil quality. While further validation is needed, this dual-trait approach presents a potential strategy for the coordinated improvement of high-OAC and high-yield phenotypes. Our findings provide molecular markers that can be applied in cultivar development and represent an initial step toward improving the precision and efficiency of peanut breeding programs.

In this study, a comprehensive multi-environment evaluation of OAC and SPP was conducted across 169 peanut accessions. Phenotypic analyses revealed a continuous distribution of OAC with a remarkable broad-sense heritability (H^2^ = 0.9634), consistent with previous findings (Yaduru et al., 2017) [3, 6, 39, 66], indicating the strong genetic control of this trait suitable for early-generation selection [29, 57]. In contrast, SPP showed low heritability (H^2^ = 0.4535), characteristic of polygenic nature and environmental sensitivity, as is typical for yield-related traits.

GWAS serve as a critical tool for dissecting the genetic architecture of complex traits by identifying trait-associated loci [25, 40, 62, 71]. In this study, GWAS analysis of 608,809 high-quality SNPs led to the identification of 32 SNPs significantly associated with OAC, with most loci concentrated on Chr9 and Chr19, regions previously implicated in fatty acid metabolism in peanut [35, 47, 63]. These loci were physically clustered near the well-characterized *FAD2* gene, a known key determinant of OAC, while also revealing novel candidate regulatory sites. The discovery of these additional sites likely reflects allelic frequency variations among different germplasm resources. Collectively, the 32 significant SNPs explained 17.65 ~ 26.23% of the phenotypic variation, reflecting the high-resolution genomic dissection enabled by dense SNP markers coverage. With respect to the origin of the favorable high-OAC alleles identified in this study, examination of the parental combinations in Supplementary Dataset S3 indicates that the OAC-associated alleles were primarily derived from the cultivar Kaixuan 016. The high frequency of OAC-enhancing alleles observed in the population is therefore attributable to the combined effects of genetic transmission from Kaixun016 and sustained artificial directional selection for high-OAC peanut cultivars.

For a set of SNPs in regions of high LD, SNPs are often co-inherited, rendering many loci redundant in terms of the genetic information they convey. To streamline analyses, a subset of representative markers, referred to as Tag SNPs, can be selected to effectively capture the underlying genetic variation within a genomic block [4, 10, 55]. Although Tag SNPs widely applied in human genetics and medicine [30, 42], this approach remains highly valuable for crop improvement. In this study, three Tag SNPs were identified through LD-based analysis. Their predictive utility was validated using multivariate regression in a 20-accession candidate population, revealing strong concordance between observed and predicted phenotypic values. Additionally, boxplot analysis of genotype–phenotype associations corroborated the discriminative power of these Tag SNPs in reliably distinguishing high- and low-OAC phenotypes, confirming their suitability for MAS in breeding programs.

GS leverages genome-wide high-density SNP markers to estimate individual breeding values by assuming at least one marker is in LD with each quantitative trait locus (QTL) governing the target trait [23]. In our study, the GS model developed for SPP yielded a heritability estimate of 0.3377, consistent with the expected polygenic architecture of complex traits in peanut [41]. According to established classifications, traits with heritability estimates below 0.20 are considered to have low heritability, those ranging from 0.20 and 0.50 moderate heritability, and those exceeding 0.50 high heritability [29]. The heritability estimate of 0.3377 obtained from our GS model therefore falls within the moderate heritability category, a range generally regarded as adequate for achieving meaningful genomic predictions in breeding programs [14]. This level of genetic control supports the application of GS for improving complex traits such as yield, for which phenotypic selection alone is often inefficient due to strong environmental influences and limited selection accuracy [13, 14, 53].

The efficiency of GS is primarily contingent upon the size, composition, and genetic relatedness of the reference population to the candidate population [15]. Maintaining consistent genetic backgrounds within the training set is crucial, as it minimizes phenotypic variation caused by genetic heterogeneity and enhances prediction accuracy by capturing true genotype–phenotype associations [14]. In this study, all 169 accessions were derived from the elite high-OAC cultivar Kaixuan016, which also served as the reference genome to ensure genetic homogeneity. The GS model achieved a prediction accuracy of r = 0.4292, measured as the Pearson correlation coefficient between predicted and observed SPP in the candidate population. This value is relatively high compared to the typical range reported for oilseed yield traits (0.3–0.4), a performance that could partly reflect optimized model construction and the relatively homogeneous genetic background of the population [31, 45]. Furthermore, our strategy of removing 30% of individuals with the lowest GEBVs demonstrated potential feasibility, mirroring successful GS-driven selection protocols in other major crops such as maize and rice [14, 54, 56].

While our integrated GWAS and GS approach show promise, several limitations should be acknowledged. First, validation of the Tag SNPs in this study was primarily conducted using a core panel of 20 accessions, which represents a relatively limited sample size. Consequently, the effectiveness and stability of these markers in broader and more diverse germplasm collections remain to be established, and further validation is required before large-scale application. Second, the GS model developed in this study was based on SPP. This choice was necessitated by the reduced reliability of plot-level yield data in certain environments due to soil-borne disease pressure. Therefore, SPP was used as a preliminary selection index to minimize non-genetic environmental effects. Future studies, where conditions permit, should incorporate standardized plot-level yield data to further improve the predictive accuracy, reliability, and practical applicability of genomic selection in breeding programs. Third, the training population used for GS was relatively small and genetically narrow, which may limit the generalizability of the model to more diverse breeding populations. Finally, the use of “Kaixuan 016” as the reference genome may introduce ascertainment bias in SNP discovery; however, a mixed linear model (MLM) was employed for GWAS analysis, in which kinship among individuals was included as a random effect, thereby effectively correcting for biases arising from population relatedness [69].

By integrating GWAS and GS methodologies, we identified key Tag SNPs associated with OAC and proposed a breeding model that has the potential to support the concurrent improvement of high-OAC and high-yield traits. Phenotypic correlation analyses revealed only a weak association between OAC and SPP (Supplementary Dataset S4), indicating that prioritizing marker-assisted selection (MAS) for OAC in early breeding stages is unlikely to increase the risk of losing favorable allelic combinations controlling yield or other agronomic traits. This supports the feasibility of the proposed stepwise breeding strategy. In the F_2_ generations, Tag SNPs can be used to rapidly fix the high-OAC genotype, thereby preventing segregation of this major-effect trait in subsequent generations. In contrast, SPP is a complex trait strongly influenced by environmental factors. Applying GS at the F_3_ generation, when genetic diversity remains relatively high, helps to reduce the risks of overfitting and unstable predictions that can arise from high relatedness and a narrow genetic base [9, 50]. Subsequent phenotypic selection in the F_4~5_ generations further validates and consolidates genomic outcomes. Overall, this integrated MAS-GS strategy is designed to achieve precise fixation of major-effect loci while efficiently accumulating favorable minor-effect alleles, thereby enhancing breeding efficiency. Although further validation across broader genetic backgrounds is warranted, this integrated approach offers a promising framework for improving complex traits improvement in oilseed crops. Looking forward, future research should focus on enhancing the predictive accuracy of GS models by expanding the size and diversity of training populations and refining statistical algorithms. In parallel, broader validation of the identified Tag SNPs across diverse peanut germplasm and the development of corresponding high-throughput molecular markers will be critical for ensuring the robustness and transferability of this approach in applied breeding programs.

## Supplementary Information

Supplementary Material 1: Dataset S1. The detailed information of the 169 Chinese peanut.

Supplementary Material 2: Dataset S2. A validation group consisting of 20 peanut materials.

Supplementary Material 3: Dataset S3. Analysis of variance for OAC across multiple environments.

Supplementary Material 4: Dataset S4. Correlation analysis between OAC and SPP across three environments.

Supplementary Material 5: Dataset S5. Significant SNP loci associated with oleic acid content traits and PVE.

Supplementary Material 6: Dataset S6. The genotypes of 169 materials corresponding to three SNP loci.

Supplementary Material 7: Dataset S7. The GEBV of 169 materials.

Supplementary Material 8: Dataset S8. The variation of GS model.

Supplementary Material 9: Dataset S9. The GEBVs of 169 accessions and their advancement rate statistics.

## Data Availability

The re-sequencing datasets of the 169 peanut germplasm resources have been deposited in the NCBI Sequence Read Archive under accession number PRJNA974180 (https://www.ncbi.nlm.nih.gov/sra/PRJNA974180).

## Abbreviations

GWAS: Genome-wide association study
GS: Genomic selection
SNP: Single nucleotide polymorphism
SSR: Simple sequence repeat
KASP: Kompetitive allele-specific PCR
OAC: Oleic acid content
SPP: Single-plant productivity
LD: Linkage disequilibrium
MAS: Marker-assisted selection
NCP: Non-centrality parameter
GBLUP: Genomic best linear unbiased prediction
BLUEs: Best linear unbiased estimates
CV: Correlation of coefficients
H^2^: Broad-sense heritability
PVE: Phenotypic variance explained
